# Risk factors for recurrent gastroesophageal reflux disease after Thal fundoplication

**DOI:** 10.1007/s00383-021-05001-1

**Published:** 2021-08-26

**Authors:** Daisuke Ishii, Hisayuki Miyagi, Masatoshi Hirasawa

**Affiliations:** Division of Pediatric Surgery, Asahikawa Medical University, 2-1-1, Midorigaoka-Higashi, Asahikawa, 078-8510 Japan

**Keywords:** Gastroesophageal reflux disease, GERD, Anti-reflux procedure, Fundoplication, Thal fundoplication, Recurrence

## Abstract

**Purpose:**

The recurrence rate of gastroesophageal reflux disease (GERD) after fundoplication has been reported to be 7–25%. We investigated the risk factors for recurrence of GERD after Thal fundoplication (TF) in our department with the aim of further reducing the recurrence rate of GERD.

**Methods:**

We retrospectively analyzed 276 patients who underwent TF for GERD at our hospital between 2000 and 2019. Retrospectively considered variables were obtained from the medical records of patients. The variables included patient characteristics, GERD severity, surgery-related factors and postoperative course.

**Results:**

The postoperative GERD recurrence rate was 5.8%. In the univariate analysis, the presence of convulsive seizures (12/4 vs. 110/150, *p* = 0.046) and the absence of a tracheostomy (0/16 vs. 53/207, *p* = 0.048) at the time of TF were significantly associated with recurrence. In the multivariate analysis, the presence of convulsive seizures at the time of TF was the only factor significantly associated with recurrence.

**Conclusion:**

The presence of convulsive seizures and the absence of a tracheostomy at the time of TF were significantly associated with GERD recurrence after TF. Active control of seizures and consideration of surgical indications, including assessment of respiratory status, are important in preventing the recurrence of GERD after TF.

## Introduction

Gastroesophageal reflux disease (GERD) has a wide spectrum of symptoms, including esophagitis (resulting from refluxed gastric acid disturbing the esophageal mucosa); aspiration pneumonia (due to the influx of reflux into the trachea); and asthma attacks due to apnea (via the vagus reflex). An association between GERD and apparent life-threatening events such as these has been reported [1]. This association is especially common among patients with severe motor and intellectual disabilities [2]. Surgical treatment should be considered for patients with persistent GERD who resist alternative medical treatment, or for those who have difficulty withdrawing from medical treatment. The most common fundoplication procedure is Nissen fundoplication (NF) [3, 4]. In the 1970s and the 1980s, NF was performed for GERD. However, since we experienced lengthy operative times for laparoscopic surgery and had many postoperative complications (such as gas-bloat syndrome) with NF, we considered alternative surgical procedures. The TF technique was therefore introduced to our surgical department in 1998 because we considered its more physiological approach to be briefer and less complicated than the NF approach. We reported [5] that the Thal fundoplication (TF) technique may be an effective and simple treatment for GERD, with few recurrences and without the complications common to NF. The advantages of TF include: (1) no surgical interference with the short gastric artery and vein, (2) brief surgery and operative time, (3) few wrap migrations, and (4) low prevalence of gas-bloat syndrome.

In most cases of GERD, fundoplication is performed to improve the patient’s quality of life (QOL), not to cure the disease. Because of this, GERD recurrence is one of the complications that surgeons most dislike. The recurrence rate of GERD after fundoplication has been reported to be 3.2–45% [6–8]. Few studies have examined the risk factors for the recurrence of GERD after TF. In the present study, we investigated the risk factors for the recurrence of GERD after TF, with the aim of further reducing the recurrence rate of GERD.

## Materials and methods

### Subject selection

The subjects included 276 patients who had undergone TF for GERD at our hospital between 2000 and 2019. The follow-up period was set at 2 years (minimum). Our department has five indicators for the surgical intervention of GERD:

1. vomiting, pneumonia, apnea, and discomfort
2. exclusion of other disorders by upper gastrointestinal imaging
3. esophageal pH-monitored regurgitation rate of 4% or more
4. a modest attempt at other, less invasive treatments
5. granting informed consent to participate in the study.

### Quantified variables

Retrospectively considered variables were obtained from the medical records of patients. The variables included age, body weight, underlying disease, convulsive seizure (for those who experienced convulsive seizures during hospital stay), preoperative tracheostomy, preoperative esophageal pH-monitored reflux rate, preoperative reflux grade (Grade 1: reflux into distal esophagus only, 2: reflux extending above carina but not into cervical esophagus, 3: reflux into cervical esophagus, 4: free persistent reflux in the cervical esophagus with a widely patent cardia, 5: reflux with aspiration into the trachea or lung) [9], operative method (laparotomy, laparoscopic or laparotomy conversion), operative time (exclusive of any time taken to perform simultaneous surgeries, such as gastrostomy), volume of bleeding, time to commencing postoperative enteral feeding, time to achieving postoperative full enteral feeding, length of hospital stay, complications (Clavien–Dindo classification) [10], and recurrence.

Postoperative GERD recurrence was defined as ‘a recurrence of symptoms (vomiting, pneumonia, apnea, and discomfort) associated with GERD, and reflux on upper gastrointestinal imaging.’

### Thal fundoplication procedure

The abdominal esophagus (at least 3 cm in length) was taped. After exposing the left and right crura of the diaphragm, the esophageal hiatus was ligated on the dorsal side of the esophagus (crural repair). Next, the fundus of the stomach was sutured to both the left wall of the abdominal esophagus and to the left crus of the diaphragm as an anchoring suture to prevent wrap migration. The stomach and left wall of the esophagus were sutured to reconstruct the His angle. Furthermore, the greater curvature of the stomach dome was sutured to both the right wall of the abdominal esophagus and the right crus of the diaphragm as an anchoring suture to prevent wrap migration. The stomach and right wall of the esophagus were sutured and wrapped over 180° anteriorly [11].

Our inclusion criteria for laparoscopic surgery are listed as follows:

- (1) body weight of 5 kg or more
- (2) no history of major abdominal surgery (patients with minor operations, such as gastrostomy, were excluded)
- (3) no severe cardiorespiratory complications

- (4) no joint contractures or severe scoliosis

### Statistical analyses

Statistical analyses were performed using EZR (Easy R, Ver. 1.41) [12]. This software, which is based on R and R commander, is freely available on the website (http://www.jichi.ac.jp/saitama-sct/SaitamaHP.files/statmed.html) and runs on Windows (Microsoft Corporation, USA). Fisher’s exact test and t-test were used for the univariate analysis. Logistic regression analysis was used for the multivariate analysis. Statistical significance was set at p < 0.05.

## Results

The results are shown in Table 1.

**Table 1:** Results

		Recurrence (+)	Recurrence (-)	Univariate analysis *p* value	Multivariate analysis		
					Odds ratio	95% CI	*p* value
*N*	*n* (%)	16 (5.8%)	260 (94.2%)				
Male/female		4/12	97/163	0.427	1.34	0.334–5.40	0.678
Age	Years	15.82 ± 17.56	16.51 ± 18.55	0.887	1.00	0.996–1.00	0.911
Body weight	kg	22.94 ± 14.62	20.55 ± 16.11	0.562	0.99	0.947–1.04	0.839
Preoperative esophageal pH-monitored reflux rate	%	14.12 ± 7.75	13.83 ± 11.05	0.919	0.99	0.931–1.06	0.824
Preoperative reflux grade	Grade	2.25 ± 0.58	1.97 ± 0.76	0.156	1.56	0.660–3.69	0.311
Convulsive seizure	±	14/2	28/232	0.026	15.32	0.013–0.63	0.010
Preoperative tracheostomy	±	0/16	53/207	0.048	1.63	Inf	0.994
Hiatal hernia	±	5/11	41/219	0.156	0.18	0.030–1.05	0.057
Esophageal atresia	±	1/15	4/256	0.260	0.09	0.206–4.22	0.163
Diaphragmatic hernia	±	1/15	6/254	0.345	0.04	0.001–1.14	0.059
Chromosomal abnormality	±	0/16	10/250		1.41	Inf	0.997
Heart disease	±	0/16	11/249		0.81	Inf	0.997
Operative procedure	Open/Lap/Conv	5/11/0	61/184/15	0.646	1.26	Inf	0.997
Operative time	min	92.88 ± 46.46	87.95 ± 36.75	0.609	0.99	0.981–1.02	0.872
Volume of bleeding	g	18.53 ± 42.14	12.08 ± 28.98	0.415	1.01	0.993–1.03	0.259
Time of restart feed	PODs	3.31 ± 0.70	3.37 ± 1.33	0.869	0.79	0.290–2.20	0.662
Time of full feed	PODs	6.38 ± 1.26	6.30 ± 1.47	0.839	1.30	0.557–3.04	0.543
Length of hospital stay	Days	9.75 ± 5.70	10.06 ± 5.81	0.835	1.02	0.898–1.16	0.753
Postoperative complication Clavien–Dindo > = 2	±	0/16	20/240	0.615	1.05	Inf	0.998

The postoperative GERD recurrence rate was 5.8% (16 cases out of 276 cases).

There were no significant differences in gender (4/12 vs. 97/163, *p* = 0.427), age (15.82 ± 17.56 vs. 16.51 ± 18.55, *p* = 0.887) and body weight (22.94 ± 14.62 vs. 20.55 ± 16.11, *p* = 0.562) when compared by recurrence status. To assess the severity of GERD, preoperative esophageal pH-monitored reflux rate (14.12 ± 7.75, 13.83 ± 11.05, *p* = 0.919) and preoperative reflux grade (2.250.58 1.97 ± 0.76, *p* = 0.156) were compared between patients with and without recurrence, but no significant difference was found.

The presence of convulsive seizures (14/2 vs. 28/232, *p* = 0.026) and the absence of a tracheostomy (0/16 vs. 53/207, *p* = 0.048) at the time of TF were significantly associated with recurrence.

For representative underlying diseases (hiatal hernia, esophageal atresia, diaphragmatic hernia, chromosomal abnormality, and heart disease), we tested for a relationship with recurrence, but found none.

No significant relationship to recurrence was found for factors related to surgery (operative procedure, operative time, and volume of bleeding). The postoperative course (time of restart feed, time of full feed, and length of hospital stay) and complications were not significantly related to recurrence either.

In multivariate analysis, the presence of convulsive seizures at the time of TF was the only factor significantly associated with recurrence.

The average time to recurrence was 19.67 ± 9.32 months after TF.

## Discussion

In this study, the recurrence rate of GERD after TF was 5.8%. The following factors were revealed to be significantly associated with recurrence: the presence of convulsive seizures and the absence of a tracheostomy at the time of TF (in univariate analysis), and the presence of convulsive seizures (in multivariate analysis). There were no correlations between recurrence risk factors and GERD severity, surgery-related factors, or postoperative course; this finding suggested that perioperative patient status is strongly associated with GERD recurrence after TF.

The incidence of recurrent GERD in patients who have undergone anti-reflux procedures has been reported to range from 3.2 to 45% [6–8], and reoperations for recurrent GERD are undertaken in 3–24% of patients [13, 14]. The mechanism of GERD recurrence is attributed to the recurrence of esophageal hiatal hernia due to mediastinal pull-up of the hilum by increased negative intrathoracic pressure caused by aspiration pneumonia and effortful breathing; and to increased abdominal pressure caused by cough, spasm, muscle tension, and intestinal peristalsis. Compounding factors include deformity of the esophageal hiatus due to progressive lateral gagging, compression of the airway due to scoliosis, and compression of the airway due to the brachiocephalic artery [15–17]. The mechanism of GERD recurrence after fundoplication was considered to be the same with both NF and TF. The risk factors for recurrence and prevention of recurrence were also considered to be the same. Although recurrence of GERD due to wrap migration has been reported in NF [18, 19], no such recurrence was observed in this study. In the reconstruction of the angle of His and the anterior wrapping of our TF technique, we always included anchoring sutures with the left and right crura. This is why wrap migration does not easily occur. The recurrence of GERD in this study was due to the recurrence of esophageal hiatal hernia or wrap disruption.

The risk factors for recurrence of GERD that have been reported are neurological impairment (NI) [13], type of procedure performed [20], age of patient at operation [21], concurrent respiratory disease [22], history of congenital esophageal malformations [23], presence of hiatal hernia [24] and the like. It has been reported that age of less than 6 years at surgery is associated with an increased risk of recurrence [16, 25]. However, in this study, there was no significant association between age and recurrence. In addition, it has been reported that fundoplication can be safely performed at age 3 years or younger [26]. Concurrent respiratory disease is a risk factor for recurrence [22, 27]. Our results are consistent with the results of previous studies. In cases of aspiration due to pharyngolaryngeal coordination disorder and repeated aspiration of saliva resulting in aspiration pneumonia, persistently high abdominal pressure is thought to lead to recurrence [27]. Fundoplication is commonly performed in patients with a history of esophageal atresia, however, the success of this surgery is limited, as reflected by an increased rate of redo fundoplication [28]. Our study included five cases of esophageal atresia, but there was only one recurrence, and no significant association was found between esophageal atresia and the risk of recurrence. Our study also included 46 cases of hiatal hernia. Among this group, there were five recurrences but there was no significant association between hiatal hernia and the risk of a recurrence.

Important components of a strategy to decrease the risk of recurrent GERD after fundoplication, include long-term management to improve recurrence factors related to systemic conditions; laryngotracheal separation (LTS) prior to fundoplication [29]; aggressive control of vomiting and retching [25]; and avoidance of postoperative esophageal dilatation [25]. Based on the results of this study, active control of seizures and consideration of surgical indications, including assessment of respiratory status, are important factors in preventing the recurrence of GERD. In this study, 80% of the recurrences occurred within 2 years of surgery, suggesting that special postoperative attention should be given in the first 2 years after surgery. LTS is difficult for patients and caregivers to accept because of the attendant loss of voice [29]. However, LTS has been shown to have dramatic advantages, such as reducing the number of suctioning sessions, eliminating aspiration when eating, and decreasing aspiration pneumonia, thereby reducing the number of hospitalizations and the risk of GERD recurrence [29].

We reported [5] that the TF technique may be an effective and simple treatment for GERD, with few recurrences and without the complications common to NF. Furthermore, we believe that TF would be a better technique if the above management could reduce the recurrence of GERD.

## Conclusion

The presence of convulsive seizures and the absence of a tracheostomy at the time of TF were significantly associated with GERD recurrence after TF. Therefore, active control of seizures and consideration of surgical indications, including assessment of respiratory status, are important in preventing the recurrence of GERD after TF.
